# The role of coronary artery calcium in allocating pharmacotherapy for primary prevention of cardiovascular disease: The ABCs of CAC

**DOI:** 10.1002/clc.23918

**Published:** 2022-09-10

**Authors:** Yash Maniar, Roger S. Blumenthal, Abdulhamied Alfaddagh

**Affiliations:** ^1^ Division of Cardiology, Ciccarone Center for the Prevention of Cardiovascular Disease Johns Hopkins University School of Medicine Baltimore Maryland USA

**Keywords:** aspirin, atherosclerotic cardiovascular disease, blood pressure, cholesterol, coronary artery calcium, diabetes mellitus, hyperlipidemia, hypertension, primary prevention, risk assessment

## Abstract

Determining optimal candidates for the numerous potential pharmacotherapies for primary prevention of atherosclerotic cardiovascular disease remains challenging. Selective use of coronary artery calcium (CAC) scoring is recommended by the 2018 and 2019 American Heart Association/American College of Cardiology Cholesterol and Primary Prevention Guidelines as a tool for refining cardiovascular disease risk assessment. A growing body of research shows that CAC has potential value in allocation of primary prevention aspirin, determining blood pressure targets and treatment intensity, the intensity of cholesterol management, and use of the more expensive medications for type 2 diabetes. We also review the literature regarding very elevated CAC scores greater than 400 or 1000 and how these scores appear to confer a risk for cardiovascular disease on par with secondary prevention cohorts.

## 1 INTRODUCTION

Cardiovascular disease has remained the leading cause of death in the United States, even during a global pandemic.^1^ Prevention of atherosclerotic cardiovascular disease (ASCVD) through modification of well‐known risk factors is therefore of paramount importance to public health. Frequently employed clinical risk scores incorporate traditional risk factors such as patient demographics, tobacco use, blood pressure (BP), diabetes, and cholesterol levels. Clinicians and cardiovascular researchers have utilized various tools to incorporate these risk factors into 10‐year ASCVD risk estimates. However, these calculators utilize surrogates for ASCVD risk rather than objective evidence of disease burden to guide management and have suboptimal sensitivity and specificity for ASCVD in large numbers of asymptomatic middle‐aged and older individuals.^2^, ^3^

Coronary artery calcium (CAC) assessment, as measured on noncontrast computed tomography (CT) scanning, quantifies the calcified plaque burden in the coronary arteries, allowing for individualized assessment of ASCVD risk. CAC scores predict ASCVD events in both retrospective and prospective cohort studies.^4^, ^5^, ^6^ In this review, we examine the role of CAC in refining ASCVD risk estimates and helping allocate medical therapies for the primary prevention of ASCVD in a more cost‐effective fashion.

The ABCDEF framework is a simplified approach to primary ASCVD prevention.^7^ The components are as follows: *A*ssessment of ASCVD risk and *A*ntiplatelet/Anticoagulant therapy, *B*P, *C*holesterol, *C*igarette smoking cessation, *D*iabetes prevention or management, *D*iet/weight, *E*xercise, and heart *F*ailure prevention and guideline‐directed treatment. We employ this model to structure the discussion of the selective use of CAC measurements in ASCVD risk assessment for allocation of aspirin, BP management, cholesterol medications such as statins, and diabetes medications—in particular GLP‐1 receptor agonizts—in primary prevention. We also review recent evidence suggesting that a category of “extremely elevated” CAC scores, above 400, and particularly over 1000, delineate a level of risk comparable to or even greater than some secondary prevention populations. We propose that these data call for more aggressive treatment of modifiable risk factors and employment of selected pharmacotherapy in this extreme risk group.

Dr. Dick Conti was always very proud that he did much of his early Cardiology work at Johns Hopkins, where he was Director of the Cardiac Catheterization Laboratory. On two separate American College of Cardiology Extended Learning interviews, he and I (Roger Blumenthal) discussed which type of patients might benefit from noncontrast cardiac CT or from CT angiography as opposed to going for coronary angiography. This paper was done in his memory and updates the impact of coronary artery calcification assessment on risk factor modification strategies.

## 2 ASSESSMENT OF ASCVD RISK USING CAC

CAC measurements improve and refine risk assessment in patients without clinical ASCVD (the primary prevention population).^8^ In fact, CAC has been shown to be a better predictor of incident ASCVD than lipid levels or traditional risk estimators.^9^ Initially, coronary plaque is predominantly noncalcified and at higher risk for rupture, and ultimately becomes more calcified with time.^10^ CAC scans only recognize and account for calcified plaque. Despite this, CAC does correlate directly with total atherosclerotic plaque burden.^11^, ^12^

A recent paper furthered the connection between CAC scores and the degree of calcified and noncalcified coronary atherosclerosis.^12^ Hollenberg and colleagues found that among symptomatic patients with suspected coronary artery disease (CAD) in the Progression of Atherosclerotic Plaque Determined by Computed Tomographic Angiography Imaging (PARADIGM) registry that the degree of obstructive CAD (≥50% stenosis) as measured on coronary CT angiography (cCTA) correlated directly with CAC scores. On mean 3.8‐year follow‐up imaging, the proportion of plaque burden that was calcified increased in patients with baseline CAC > 100. Moreover, in subjects with CAC scores of ≥400, there was disproportional growth in the calcified plaque with a volumetric increase 16 times that of noncalcified plaque.

Ultimately, coronary plaque burden does confer an increased risk for adverse cardiac outcomes. A CAC score of 0 has a strong negative predictive value for clinical cardiovascular events and death in primary prevention populations over the next 5–10 years.^13^ This “power of zero” has led to the American Heart Association (AHA)/American College of Cardiology (ACC) clinical practice guidelines adopting selective use of CAC as a tool to help de‐risk patients.^14^ On the other hand, CAC scores above 300 are associated with much higher risks of ASCVD events than scores of 0 or those that are below the 25th percentile for one's age and gender.

In a cohort study of 4129 middle‐aged adults without known ASCVD, the relative risk (RR) of nonfatal myocardial infarction (MI) or death from coronary heart disease (CHD death) was much greater in those with elevated CAC (3.98, CAC 100–399; 9.94, CAC ≥ 400) compared to those with CAC of 0 at 5‐year follow‐up.^6^ Another study from the Multi‐Ethnic Study of Atherosclerosis (MESA) cohort found that CAC strongly predicts ASCVD risk regardless of sex, age group, or race/ethnicity.^15^

Higher CAC scores ≥1000 signify a very elevated risk of ASCVD, comparable to secondary prevention cohorts.^16^, ^17^ A 2021 analysis of participants in MESA found that adults with baseline CAC ≥ 1000 had a significantly elevated risk of ASCVD events, CHD events, and all‐cause mortality when compared to both those with CAC of 0 (RR 4.71, 7.57, and 1.94, respectively) and CAC 400–999 (RR 1.65, 1.66, 1.34) after a mean follow‐up of 13.6 years.^17^ Furthermore, the CAC ≥ 1000 group had more extensive extra‐coronary calcification, significantly increased CAC area, and increased risk of noncardiovascular events (defined as cancer, chronic kidney disease, pneumonia, chronic obstructive pulmonary disease, deep vein thrombosis/pulmonary embolism, dementia, and hip fracture).

Altogether, this data calls for an adjustment of the primary versus secondary prevention paradigms, as these two groups may overlay one another based on underlying CAC. This holds treatment implications that begin with a focus on intervention on modifiable risk factors. A 2013 retrospective study showed that CAC ≥ 1000 is associated with higher rates of hypertension, hypercholesterolemia, diabetes mellitus, and obesity than those with CAC 400–999, with high BMI and age found to be independent risk factors for CAC ≥ 1000 on multivariate analysis.^18^

Tobacco use also portends an elevated risk of extreme CAC and is associated with poor outcomes among those with extreme CAC. A cohort study of 10 377 asymptomatic individuals without known CAD found that current smokers carried an increased prevalence of higher CAC scores at all strata (11–100, 101–400, 401–1000, and >1000) compared with nonsmokers, and a progressively elevated increased risk of all‐cause mortality compared with nonsmokers in the same CAC score category (RR 2.41, 3.35, 5.59, and 10.93, respectively) at 5‐year follow‐up.^19^ Clinically, therefore, an elevated CAC score should serve as a strong motivator to the current smoker or obese patient to adequately address their underlying risk factors and should push the clinician to more aggressively treat these medical problems, with both lifestyle and pharmacologic therapies.

## 3 ALLOCATION OF ASPIRIN FOR PRIMARY PREVENTION WITH CAC

Despite the highly prevalent use of aspirin for primary prevention of ASCVD historically, new data has called into question the overall benefit of aspirin given the associated increased risk of gastrointestinal hemorrhage.^20^ In response, the 2019 ACC/AHA Primary Prevention of ASCVD guideline changed the recommendation for low‐dose aspirin from Class I to Class IIb in select adults at elevated ASCVD risk but not elevated bleeding risk.^14^

**Figure 1:**
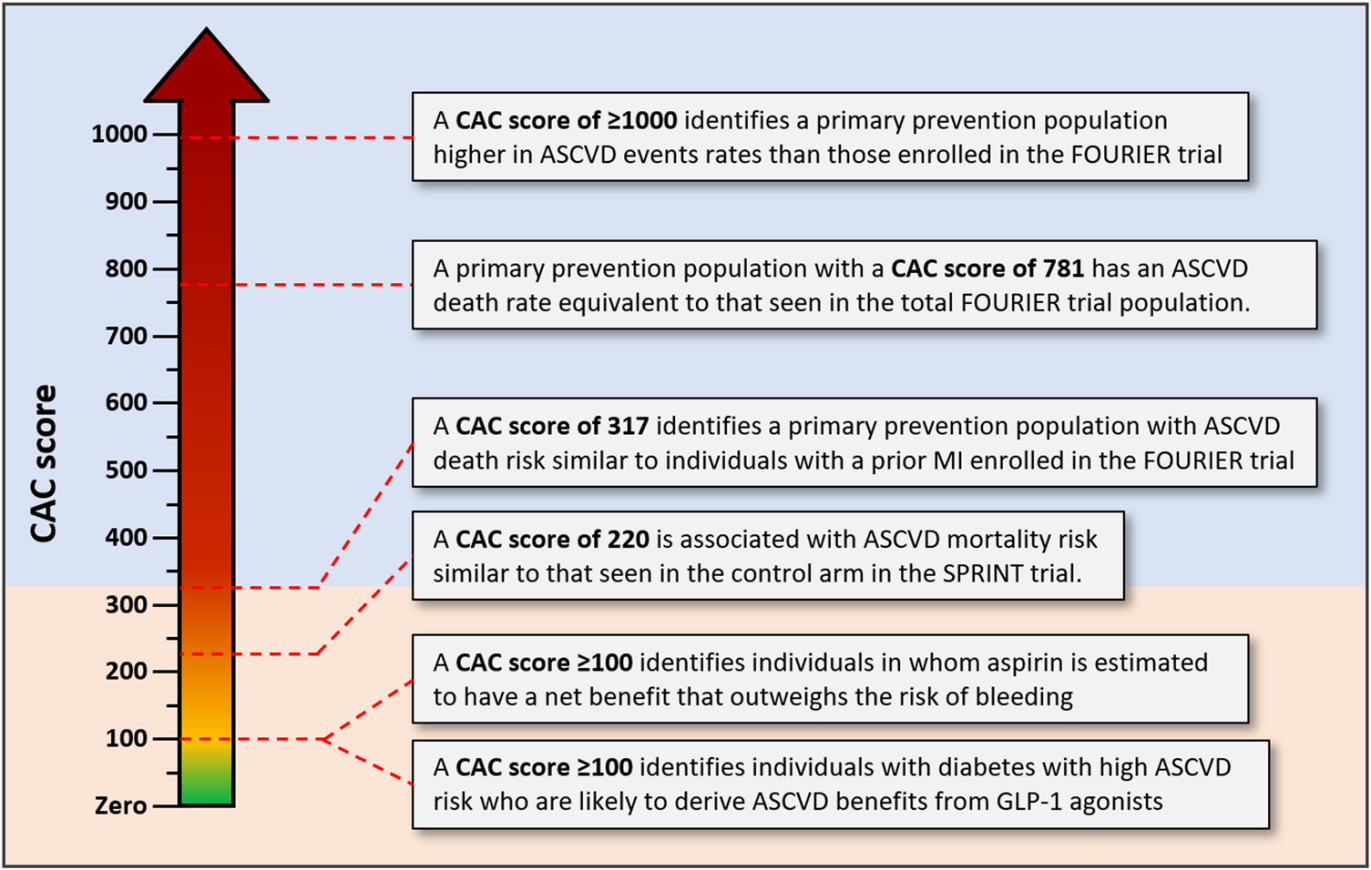
Advanced CAC scores identifies a population with very elevated ASCVD risk (equivalent to that in secondary prevention cohorts) who may benefit from aggressive preventive treatments. ASCVD, atherosclerotic cardiovascular disease; CAC, coronary artery calcium; SPRINT, Systolic Blood Pressure Intervention Trial.

A 2014 study of 4229 aspirin‐naïve subjects from the MESA cohort investigated the role of CAC in identifying optimal candidates for primary prevention aspirin.^21^ Five‐year number needed to treat (NNT) estimates were calculated using a threshold of 18% RR reduction (RRR) in coronary events, and 5‐year number needed to harm (NNH) estimates were calculated using major bleeding risk from prior studies. Individuals with CAC ≥ 100 were determined to have an estimated likely net benefit with aspirin (low NNT, high NNH), while those with CAC of 0 had an estimated net harm. After consideration of these data, the Society of Cardiovascular CT recommended the strong consideration of aspirin in most patients with CAC > 100 who do not have elevated bleeding risk (Figure 1).^22^

A subsequent 2020 analysis of 3540 MESA participants who were not at elevated bleeding risk was performed after the release of the updated ACC/AHA guidelines, strengthening the prior study's findings by using updated meta‐analysis data and observed bleeding rates to generate NNH.^23^ The 5‐year NNT was created from a 12% RRR in ASCVD events, and the 5‐year NNH was created from a 42% RR increase in major bleeding events. Again, CAC ≥ 100 identified subjects who likely would derive a net benefit from aspirin, and subjects with CAC of 0 would likely have net harm, irrespective of the subjects' underlying ASCVD risk.

Finally, another paper, including 2191 subjects from the Dallas Heart Study cohort (DHS), also estimated that aspirin likely provided a small net benefit for those with matched CAC ≥ 100 with low bleeding risk and an ASCVD risk >5% per the pooled cohort equation (PCE), and probably net harm (increased major bleeding events) in all individuals with higher bleeding risk.^24^ CAC scores correlated with increased major bleeding and ASCVD events over a mean 12.2‐year follow‐up. While prospective data are still lacking, these studies indicate a possible role for selective use of CAC in the guideline recommendations for the allocation of aspirin for primary prevention and confirm the relatively poor test characteristics of risk scores in this domain.

## 4 BLOOD PRESSURE MANAGEMENT AND CAC

The link between elevated BP and CAC has been well‐studied. Elevated CAC is a risk factor for hypertension, and vice‐versa; notably, the link between elevated CAC and adverse cardiovascular outcomes has been established in hypertensive patients.^25^ The decision of when to initiate medications for the treatment of newly diagnosed essential hypertension is not yet settled. The 2017 ACC/AHA Hypertension Guideline recommends a quantitative assessment of ASCVD risk to guide hypertension management and set BP goals.^26^ Management of Stage 1 hypertension in low‐risk adults (no ASCVD or 10‐year risk estimate <10%) begins with nonpharmacologic therapy.^27^

CAC has been proposed as a tool to assess and refine ASCVD risk in patients with hypertension, to better gauge who may benefit from early initiation of BP medications. A pooled cohort analysis of 6461 patients not on antihypertensive treatment from the Jackson Heart Study, Coronary Artery Risk Development in Young Adults (CARDIA), and MESA compared the risk of incident ASCVD events (heart failure, stroke, or CHD) in adults stratified by degree of hypertension and by CAC score.^28^ Over a median 8.5‐year follow‐up, the estimated 10‐year NNT for BP was found to be lower in both BP treatment groups in those with nonzero CAC (36 for the elevated BP/low‐risk Stage 1 hypertension group and 22 for high‐risk Stage 1 hypertension/Stage 2 hypertension group) compared to those with CAC of 0 (160 and 44, respectively).

Weinberg et al.^25^ proposed an algorithm incorporating CAC scores for patients ages 40–75 without compelling indications for up‐front pharmacologic management (certain comorbidities, systolic BP (SBP) ≥ 160, or diastolic BP ≥100) who have intermediate 10‐year ASCVD risk scores (5%–20%). Their recommendation, based on expert opinion and observational studies, is to start with up‐front therapy in those with lifestyle changes in those with CAC of 0, medications for patients with CAC ≥ 100, and a gray zone of CAC 1–99 where a CAC threshold of 10 favors medication initiation. The algorithm also suggests the same CAC thresholds to determine whether to target a more liberal (140/90) or a stricter (130/80) BP goal.

A similar CAC‐guided approach could be utilized to help guide lifestyle management in patients with Stage 1 hypertension, younger patients, or patients with difficulty adhering to medications. For instance, if CAC was obtained in an adult with Stage 1 hypertension for a different indication (e.g., to inform the decision to start a statin in a patient with intermediate ASCVD risk), an elevated score should serve as a trigger to more aggressively pursue lifestyle changes pursuant to lowering BP. Prospective trials are needed to appraise clinical outcomes of hypertension management guided by CAC.

The optimal pharmacologic management of hypertension takes into account many factors, including patient ability and willingness to change modifiable lifestyle factors, known medical comorbidities, risk of hypotension, medication conflicts, and assessment of ASCVD risk. The role of CAC in allocating antihypertensive therapy intensity was examined in a 2017 study of 3733 subjects from the MESA cohort, stratified by SBP and ASCVD risk estimate.^29^ Mean 10‐year NNT estimates (of ASCVD events or heart failure hospitalizations) varied significantly with CAC score in subjects with SBP < 160, especially in those also with 10‐year risk <15%, but not in those with SBP > 160, who had higher event rates regardless of CAC. These results raise the possibility of using CAC to stratify hypertensive patients to more or less aggressive BP treatment (a traditional goal of <140 or a more intensive goal of 120mmHg in adults with an estimated ASCVD risk of 5%–15%).

A recent paper investigated the utility of CAC for risk stratification among 16 167 hypertensive subjects of the CAC Consortium cohort.^30^ Within this group, 6375 participants were classified as similar to the population of the landmark Systolic Blood Pressure Intervention Trial (SPRINT) with a Framingham risk score >15% and age >50.^31^ At a mean follow‐up of 11.6 years, the risk of CHD and ASCVD mortality was associated with the CAC score, across 10‐year ASCVD risk categories as calculated by the PCE, in both the total study and the “SPRINT‐like” population. Modeling analyses showed that a CAC score of 220 was associated with the ASCVD mortality rate seen in SPRINT (Figure 1). This threshold, therefore, may be reasonable for identifying candidates for aggressive antihypertensive therapy.

## 5 CHOLESTEROL MANAGEMENT USING CAC

The AHA/ACC/Multisociety Cholesterol Guideline stated that CAC can be considered in allocation of statins for primary prevention.^32^ They assign an IIa recommendation to selective use of CAC scoring for patients with a 10‐year ASCVD risk of 5%–20% to guide decisions regarding initiation of treatment. Due to the prognostic “power of zero,” indicating a low short‐term risk of ASCVD events, a CAC score of 0 among these patients allows for the deferral of statin therapy. On the other hand, initiation of statin therapy is reasonable with detectable CAC and especially for those with CAC scores >75th percentile for their age and gender or an absolute score of >100. Notably, CAC has been shown to predict ASCVD for patients on statins and those off statins.^15^

In the previously referenced MESA study examining CAC scores over 1000, a logarithmic model was generated relating annualized rates of major adverse cardiovascular events (MACE) to CAC scores.^17^ A CAC score of 1000 in primary prevention patients showed similar MACE rates upon comparison with the cohort of secondary prevention patients in the Further cardiovascular Outcomes Research with proprotein convertase subtilisin/kexin type 9 (PCSK9) Inhibition in subjects with Elevated Risk (FOURIER) trial (Figure 1).^33^ The annualized MACE rate of the FOURIER secondary prevention cohort was equal to that of patients in the MESA cohort with of CAC score of 900.

A subsequent study used data from the CAC Consortium cohort, specifically including patients who were over 50 years of age with a 10‐year ASCVD risk ≥7.5%, to develop a regression model relating annual ASCVD mortality to CAC scores.^34^ The risk of ASCVD mortality of the FOURIER secondary prevention cohort was equivalent to that of primary prevention patients with CAC scores of 775–900. These studies highlight a potential role for a re‐evaluation of low‐density lipoprotein goals and more aggressive lipid‐lowering in these very high‐risk patients.

A multifaceted treatment approach may be warranted, involving medications such as ezetimibe and possibly a PCSK9 inhibitor in those with severe CAC. A retrospective longitudinal study of patients undergoing coronary CTA found that progression of CAC on follow‐up imaging was reduced in those prescribed a PCSK9 inhibitor in addition to statins (14.3% annually), compared to statin monotherapy (29.7%).^35^ However, this study included patients who had symptoms and EKG changes suspicious for CAD, and so the results may not be generalizable to the primary prevention population. Additionally, the sample size was small, with only 120 subjects, none of whom had CAC > 1000. Further research is needed to elucidate the role of CAC in allocating advanced, nonstatin lipid‐lowering therapies, especially among the very high‐risk population with CAC > 1000.

## 6 DIABETES AND PRIMARY PREVENTION USING CAC

Although diabetes mellitus is a well‐established ASCVD risk factor, there remains significant individual risk variability among those with diabetes. CAC has an important role in improving risk stratification in patients with diabetes.^36^ However, due to insufficient prospective data, the “power of zero” is not recommended to routinely “de‐risk” patients with diabetes aged 40 years and older to avoid statin therapy. Currently, guidelines recommend moderate‐intensity statin therapy for those with type 2 diabetes aged 40–75, and high‐intensity statin therapy for those with diabetes and multiple ASCVD risk factors.^14^ That being said, a CAC score of 0 or a low percentile for one's age and gender can help guide shared decision‐making in patients with statin‐associated symptoms, polypharmacy, or a strong preference to forgo statins.

With regard to glycemic therapies, the glucagon‐like peptide 1 receptor agonizts (GLP1‐RAs) and sodium‐glucose cotransporter 2 (SGLT2) inhibitors have both shown reductions in ASCVD events in patients with diabetes.^36^ A systematic review and meta‐analysis of seven trials of GLP1‐RAs in patients with diabetes found significant reductions in MACE and each component outcome (cardiovascular death, MI, and stroke).^37^ One of these randomized placebo‐controlled trials, the 2019 REWIND trial, showed a statistically significant reduction of ∼11% in MACE in patients ages >50 with type 2 diabetes randomized to weekly dulaglutide compared to placebo at after a mean follow‐up of 5.4 years.^38^ Importantly, this trial showed equal relative reductions in both the 6221‐subject primary prevention cohort as well as the secondary prevention cohort. At the same time, the NNT was much lower in the secondary prevention cohort, with a greater absolute reduction. This may indicate that the costly GLP1‐RAs may not be cost‐effective at current prices solely for the primary prevention of ASCVD unless they have significant subclinical coronary atherosclerosis.

A pooled cohort analysis of 1125 adults with diabetes without established ASCVD from MESA, CARDIA, and DHS studied the role of CAC in the allocation of GLP1‐RAs for the prevention of MACE.^39^ The 5‐year NNT for patients with a CAC of 0 was three times greater than those with CAC > 100. In fact, subjects with CAC > 100 had a significantly lower 5‐year NNT than the studied population eligible for GLP1‐RAs by traditional guidelines. Currently, GLP1‐RAs are recommended for diabetics with cardiovascular risk factors who have an elevated hemoglobin A1c despite optimal first‐line therapy, including metformin, dietary counseling, and exercise. In the absence of other indications for these medications, such as heart failure and obesity, CAC may be a useful tool to triage the prescription of GLP1‐RAs for primary prevention in adults with diabetes to those who may derive the greatest benefit (e.g., those with high amounts of subclinical coronary atherosclerosis) (Figure 1).

In addition, some of the aforementioned research on CAC scores also applies to patients with diabetes. Very high‐risk patients with CAC ≥ 400 or ≥1000 may derive a greater benefit from lipid‐lowering therapy, potentially with the addition of nonstatin medications such as ezetimibe in those treated with a high‐intensity statin, to achieve much lower low‐density lipoprotein cholesterol goals.^36^ The utility of CAC in allocating SGLT2 inhibitors for primary prevention of ASCVD in diabetes remains unexplored to date. CAC can help stratify those with diabetes into higher and lower risk groups, to help target more intensive interventions to those most likely to benefit.^8^

## 7 CONCLUSION

CAC scoring has an established role in refining ASCVD risk assessment and growing potential as a tool to allocate a variety of pharmacotherapies in primary prevention. Clinicians and patients may decide to be less aggressive in the use of drug therapy if no CAC is present; by contrast, above‐average amounts of CAC for one's age support a more aggressive approach. Very elevated CAC scores above 1000 among patients without established ASCVD may signify a population that benefits from more aggressive prevention treatments.

## CONFLICT OF INTEREST

The authors declare no conflict of interest.

## Data Availability

Data are not applicable to this review article as no new data were created.
